# Comparison of primary total hip replacements performed with a direct anterior approach versus the standard lateral approach: perioperative findings: Reply to Letter by Sanjay Meena

**DOI:** 10.1007/s10195-012-0192-0

**Published:** 2012-04-25

**Authors:** Vincenzo Alecci, Maurizio Valente, Chiara-Martina Pellegrino

**Affiliations:** 1 Orthopaedics and Traumatology Unit, Ospedale San Polo, via Galvani 1, 34074 Monfalcone, GO Italy; 2 Anesthesia and Intensive Care Unit, Ospedale San Polo, Monfalcone, GO Italy

Thank you for your positive comments to our article.

We implanted an uncemented prosthesis in both group A (198 patients: Bauer’s standard lateral approach and spinal or general anesthesia) and group B (221 patients: minimally invasive direct anterior approach and general anesthesia). We always use hemispherical cups coupled with different types of stems. In the majority of cases we used straight stems followed by anatomical stems, while lately we have started to use short stems that are expressly designed for minimally invasive access. Having said that, cemented implants can also be used with a minimally invasive direct anterior approach, as reported by expert authors such as Matta and Rachbauer [1, 2]. We use cemented implants in cases of femoral neck fracture that are treated with hemiarthroplasty.

Both groups followed the same rehabilitation protocol and had the same rehabilitation goals. Complete functional recovery was achieved earlier and therefore the length of hospital stay (LOS) was reduced in group B. Patients in group A achieved a sufficient degree of autonomy to allow discharge on day 10 on average, and started walking without crutches around week 8. Patients in group B achieved the same goals at day 7 and walked autonomously in week 2. This difference is due, in our opinion, to the tissue sparing associated with the minimally invasive anterior approach.

Analgesic protocols were highly heterogeneous; they depended on the patient’s characteristics and the preferences of the anesthesiologist. This variability means that we cannot evaluate the effectiveness of each individual protocol in correlation with NRS and type of surgery. We have no data on the actual analgesic drug requirements. Mainly i.v. drugs (opioids + NSAID) were administered (via elastomeric pumps) in both groups (Table 1).

**Table 1:** NRS on postoperative day 1

	Group A	Group B	
NRS (mean)	2.5	1.4	*p* < 0.05
Analgesic protocol			
Meperidine + NSAID	80.3 %	42.5 %	
Morphine + NSAID	13.7 %	2.3 %	
Tramadol + NSAID	2.5 %	20.8 %	
Oxycodone + paracetamol	0	25.8 %	
Other	3.5 %	8.6 %	

Statistical analysis: NRS: Pearson’s* χ* ^2^ test
